# Reversal of Multidrug Resistance by the Chinese Medicine Yiqi Jianpi Huaji Decoction and the Mechanism of Action in Human Gastric Cancer SGC7901/VCR Cells

**DOI:** 10.1155/2015/390812

**Published:** 2015-02-02

**Authors:** Wei-Bing Li, Yang Li, Chen Yu, Yong-Ming He

**Affiliations:** ^1^Department of Integrated Traditional and Western Medicine, Jiangsu Cancer Hospital, Baizi Ting, No. 42, Nanjing, Jiangsu 210000, China; ^2^Department of Radiation Oncology, Cancer Center, Sun Yat-sen University, Guangzhou, Guangdong 510006, China

## Abstract

Yiqi Jianpi Huaji Decoction (YJHD), a traditional Chinese medicinal formula composed of twelve ingredients, has recently been reported to have a good clinical curative effect. The purpose of the present study was to evaluate the effects of YJHD on SGC7901/VCR gastric cancer cells and to elucidate the possible mechanism of action. First, the effects of a low dose of YJHD in combination with chemotherapeutic agents on SGC7901/VCR cells were assessed using the CCK-8 assay and flow cytometry, and the effects of YJHD on genes and proteins involved in drug resistance (MDR1, MRP, TUBB3, STMN1, and TS) were evaluated. Furthermore, transfection of SGC7901/VCR cells with siRNAs targeting these genes inhibited their expression, and the efficacy of vincristine against the cells was dramatically improved in vitro when these genes were silenced. These results demonstrate that low-dose YJHD inhibited cell proliferation, induced apoptosis, reversed MDR, and increased sensitivity to chemotherapeutic agents in vitro by downregulating P-gp, MRP, TUBB3, and STMN1 expression. MDR can be reversed by siRNAs targeting genes involved in MDR, and this strategy for cancer treatment should be evaluated in future studies.

## 1. Introduction

Stomach cancer was the cause of 738,000 deaths in 2008, corresponding to approximately 9.7% of the total number of cancer-related deaths [1], and the incidence and mortality rates for stomach cancer are significantly higher in China than in the rest of the world [2]. The major factors responsible for the increased mortality and morbidity associated with gastric cancer are the progression of the disease and failure of chemotherapy caused by multidrug resistance (MDR) [3] via the overexpression of the MDR1 and MRP genes [4]. The major cause of MDR in tumor cells is the overexpression of a membrane-bound protein, P-glycoprotein (P-gp), and other members of the adenosine triphosphate (ATP) binding cassette (ABC) transporter superfamily [5], which translocate a substrate from the intracellular compartment to the extracellular compartment, leading to a reduced intracellular concentration of the substrate and resistance to antineoplastic drugs [6]. The overexpression of P-gp and MRP is associated with a poor prognosis. In normal gastric tissue, P-gp and MRP mRNAs are either not present or are only slightly elevated [7], whereas P-gp and MRP overexpression is associated with MDR in gastric cancer [8–10].

However, several other mechanisms are also involved in the development of MDR in tumor cells, including alterations in drug targets (e.g., TUBB3, STMN1, and TS), the activation of detoxifying systems, the interruption of signaling pathways, and alterations in regulators involved in cell cycle control [6], and the above genes exhibit chemotherapy-related effects. A number of studies have confirmed that a high TS expression level contributes to the resistance to 5-Fu and a poor clinical outcome [11]. Several studies using drugs that target microtubules have also found that *β*-tubulin III (TUBB3) and stathmin 1 (STMN1) are directly related to the efficacy of chemotherapy [6]. Additionally, elevated TUBB3 expression is related to vincristine resistance and a short survival time [7, 8], and STMN1 affects the formation of the mitotic spindle, promoting microtubule depolymerization or preventing microtubule polymerization [8].

Herbal medicines are an important source of novel agents with pharmaceutical potential [9]. Modern pharmacological experiments have led to the use of herbal medicines for complementary and alternative therapy, and this approach has become increasingly popular, particularly in cancer patients who exhibit resistance after several chemotherapy treatments [10, 12]. Considering the narrow therapeutic window of chemotherapeutic agents, synergistic or additive interactions may improve the outcome of a therapy and reverse long-term chemotherapy drug resistance. As a result of the variation in adjuvant components, each formula can have a different name, and the precise mechanisms of action of these formulae also remain to be addressed in studies investigating their clinical efficacy. One such formula, Yiqi Jianpi Huaji Decoction (YJHD), is currently under study in our laboratory. YJHD is a prescribed Chinese complex that contains the following twelve ingredients:* Codonopsis pilosula, Astragalus membranaceus, Atractylodes macrocephala koidz, Radix angelica, sinensis Radix paeoniae alba, *Tangerine peel,* Pinellia tuber, Zedoary, Rhizoma sparganii, Salvia chinensis*, licorice, and* Oldenlandia diffusa*. YJHD shows good effect in clinical efficacy, and three components (*Salvia chinensis*,* Zedoary*, and* Oldenlandia diffusa*) exhibit potential anticancer activity and restore the sensitivity of MCF-7/ADR and A549/Taxol cells, modulating the MDR phenotype and the function of P-gp in vitro [13, 14].* Oldenlandia diffusa* extracts exert antiproliferative effects and induce apoptosis in human breast cancer cells [15]. Additionally, the individual use of other YJHD components has been reported in China and was found to strengthen the immune system, improve appetite, and act as antiangiogenesis and as an anticonvulsant [16]. However, the potential role of this formula in cancer therapy has not yet been clearly addressed by modern science. To further elucidate mechanisms that may be involved in the interaction between YJHD and chemotherapeutic agents, we also investigated apoptosis levels and the expression of chemotherapeutic agent resistance-related genes in gastric cancer cells after treatment with YJHD and chemotherapeutic agents singly and in combination.

RNAi is a fundamental cellular mechanism for silencing gene expression and can be used for the development of new drugs [17]. Accordingly, this study focused on the downregulation of MRP, MDR1, TUBB3, and STMN1 expression by siRNA. After transfection of siRNAs, silencing at the protein level was demonstrated by western blot analysis. Furthermore, we also examined the in vitro effects of siRNA-mediated MRP, MDR1, TUBB3, and STMN1 suppression on the chemotherapeutic sensitivity of human SGC7901/VCR cell lines.

## 2. Materials and Methods

### 2.1. Preparation of YJHD Extract

All of the crude components, including 15 g* Codonopsis pilosula*, 15 g* Astragalus membranaceus*, 10 g* Atractylodes macrocephala koidz*, 10 g* Radix angelica sinensis*, 10 g* Radix paeoniae alba*, 6 g* Tangerine peel*, 10 g* Pinellia tuber*, 10 g* Rhizoma sparganii*, 10 g* Zedoary*, 30 g* Salvia chinensis*, 5 g licorice, and 30 g* Oldenlandia diffusa*, were purchased from the Jiangsu Provincial Hospital of Traditional Chinese Medicine (Nanjing, Jiangsu Province, China). First, a mixture of YJHD was homogenized with a Waring blender, then soaked in 10 L (10-fold of the plant) double-distilled water for 2 h, and boiled for 1 h. The cooled decoction was filtered through two layers of cotton gauze. The filtrates obtained from three cycles of the procedure were combined, and the filtered solution was concentrated into a residue in a vacuum evaporator and distilled into a liquid. The yield of the aqueous extract was 2.1 g/mL based on the original amounts of the herbal ingredients.

The extract was reconstituted with sterile distilled water to prepare working solutions with final concentrations of 6, 4, 3, 2, 1, 0.5, and 0.25 mg/mL for the treatment of cancer cells. The quality control of the YJHD preparation, including the verification of the correct plant components, origin of production, and growth, harvesting, and processing, was performed according to the guidelines defined by Nanjing Herb Pharmaceutics Ltd. (Nanjing, Jiangsu Province, China).

### 2.2. Cell Lines and Cell Culture

The human gastric cell line SGC7901 was kindly provided by the center laboratory of Jiangsu Cancer Hospital, Nanjing, China. The human MDR gastric cancer cell line SGC7901/VCR was purchased from the Shanghai Institutes for Biological Sciences, Chinese Academy of Sciences (Shanghai, China). The cells were propagated in RPMI 1640 medium (GIBCO BRL, CA, USA) supplemented with 10% bovine serum, penicillin (100 U/mL), and streptomycin (100 *μ*g/mL) at 37°C in a water-saturated atmosphere with 5% CO_2_. For the SGC7901/VCR cell line, the medium contained an additional 1 *μ*g/mL vincristine (Shenzhen Main Luck Pharmaceuticals).

### 2.3. Drugs

5-Fluorouracil (5-Fu) was supplied by Jiangsu Hengrui Medicine Company (Jiangsu, China). Vincristine sulfate (VCR) was purchased from Shenzhen Main Luck Pharmaceuticals (Shenzhen, Guangdong, China). The dilutions of all of the reagents were prepared fresh before each experiment. An Annexin V-FITC flow cytometry detection kit was purchased from Nanjing KeyGEN Biotech. Co, Ltd. (Nanjing, Jiangsu, China).

### 2.4. Analysis of Cell Viability

The CCK8 assay was used to measure cell viability. Exponentially growing SGC7901/VCR cells (100 *μ*L, 5 × 10^4^ cells/mL) were seeded into 96-well plates (Corning Incorporated, Corning, NY, USA). After 24 h, the cells were cultured in an RPMI-1640 medium containing 10% FBS and then treated with VCR or 5-Fu at concentrations ranging between 40 and 0.625 *μ*g/mL as well as YJHD at concentrations between 6 and 0.5 mg/mL. Then, we examined the combined effect of a low-dose YJHD and VCR or 5-Fu. The treatments were repeated three times at each concentration. The plates were then incubated in a humidified incubator (Sanyo, Osaka, Japan) at 37°C and 5% CO_2_ for 48 h. Subsequently, 2 h prior to measuring the absorbance, 10 *μ*L of CCK-8 solution was added to each well. The optical density was measured at an absorbance of 450 nm using a microplate reader (BioTek Instruments, Winooski, VT, USA). The rate of inhibition of cellular proliferation was calculated using the following formula: cellular proliferation inhibition rate = (1 − mean A_450_ of experimental group/mean A_450_ of control group) × 100%. We chose to evaluate the chemotherapy drugs with the highest fold reversal levels.

### 2.5. Flow Cytometry

The effects of the evaluated concentrations of YJHD and chemotherapeutic drugs on apoptosis were determined by flow cytometry. The cells were seeded at 3 × 10^5^ cells/well in six-well plates and incubated overnight. A low dose of YJHD (0.5 mg/mL) was added in combination with 0.625 *μ*g/mL–2.5 *μ*g/mL of 5-Fu, and the plates were incubated for 48 h. Dimethylsulfoxide-treated cells (0.1%) served as the control. The cells were collected and washed twice with 4°C PBS. The cells were then added to 500 *μ*L of binding buffer, and 5 *μ*L of Annexin V/FITC and 5 *μ*L of propidium iodide (PI) were then mixed. The experiments were performed in triplicate.

### 2.6. Cell Cycle Analysis

For the cell cycle analysis, SGC7901/VCR cells were cultured in 6-well plates (at least 3.0 × 10^5^ cells/well) in an RPMI-1640 medium containing 10% FBS and 100 U/mL of penicillin and streptomycin for 24 h and then treated with a low dose of YJHD (0.5 mg/mL) and 0.625 *μ*g/mL of 5-Fu for 48 h. The cells were washed, collected, and fixed using 70% ethanol. Following that step, the cells were treated with TrisHCl buffer (pH 7.4) containing 1% RNase A (KeyGen Biotech Co., Ltd.) and stained with PI. Flow cytometry (FACSCalibur; Becton-Dickinson) was performed to determine the distribution of cells with varying DNA contents. Multicycle DNA content data were analyzed using cell cycle analysis software (KeyGen Biotech Co., Ltd.).

### 2.7. RNA Extraction and Real-Time Reverse Transcriptase-PCR

Total RNA was extracted using Trizol (Invitrogen) according to the manufacturer's instructions. For mRNA analysis, real-time PCR was performed using the Power SYBR green PCR master mix (Applied Biosystems) with an ABI 7500 series PCR machine (Applied Biosystems), and the data were normalized to GAPDH expression and then further normalized to the negative control, unless otherwise indicated. Custom primers for* TUBB3*,* STMN1*,* TS*,* MDR1*, and* MRP* were synthesized by Ruian Biotech (GAPDH forward primer 5′-CCATGGAGAAGGCTGGGG-3′ and reverse primer 5′-CAAAGTTGTCATGGATGACC-3′; TUBB3 forward primer 5′-GCCTGACAATTTCATCTTT-3′ and reverse primer 5′-TCACACTCCTT CCGCACCA-3′; STMN1 forward primer 5′-AGAATACACTGCCTGTCGCTT G-3′ and reverse primer 5′-AGGCACGCTTCTCCAGTT-3′; TS forward primer 5′-ACCTGAATCACAATC GAGCCA-3′ and reverse primer 5′-TTG GATGCGGATTGTACCCT-3′; MDR1 forward primer 5′-CACGTCAGCCTTGGA CACAGA-3′ and reverse primer 5′-CAA TGACTCCATCATCGAAACCAG-3′; MRP forward primer 5′-TCTCTCCCGACATGACCGAGG-3′ and reverse primer 5′-CCAGGAATATGCCCCGACTTC-3′).

### 2.8. Western Blot Analysis

The proteins in the SGC7901/VCR cell culture supernatant were analyzed directly for STMN1, TUBB3, MDR1, MRP, and TS detection, and cell extracts were prepared in a suitable lysis buffer. In brief, each sample was mixed with loading buffer, separated by electrophoresis at a constant voltage and electrotransferred onto PVDF membranes (Millipore, Billerica, MA, USA). The membranes were incubated with a primary antibody at 4°C overnight, washed three times with TBS-T buffer, and incubated with a secondary antibody conjugated to horseradish peroxidase (Cell Signaling Technology, CST, Beverly, MA, USA) for 1 h. After three washes, the blots were visualized using the enhanced chemiluminescence method (Millipore). Rabbit monoclonal antibodies against STMN1, TUBB3, MDR1, MRP, and TS were purchased from Abcam (Abcam, Cambridge, UK).

### 2.9. Transfection with siRNAs

Cells were seeded in 6-well plates and after, 24 h of growth, were then transfected with the indicated siRNAs (MDR1, MRP, STMN1, and TUBB3) using Lipofectamine RNAiMAX Reagent according to the manufacturer's protocol. Silencing was examined at 24 h after transfection by western blotting. The siRNA-transfected cells (5 × 10^4^ SGC7901/VCR cells/mL) were plated in 96-well plates and treated with various concentrations of the test agents. After 48 hr, the IC_50_ values for vincristine in these cells were determined by the CCK-8 assay. The siRNAs were synthesized by RiboBio (Guangzhou). The target sequences were as follows:* MDR1* siRNA: 5′-AA GACATGACCAGGTATGCCT-3′;* MRP* siRNA: 5′-CGAUGAAGACCAAGAC GUAUU-3′;* TUBB3* siRNA: 5′-UCUCUUCAGGCCUGACAAUTT-3′;* STMN1* siRNA: 5′-CAGTGGATTGTGTAGAGTGTA-3′. A negative control, 5′-UUCUCCGAACGUGUCACGUTT-3′, was also obtained from Dharmacon.

### 2.10. Statistical Analysis

All of the data are expressed as the mean ± SD. The results were analyzed using *t*-tests and analyses of variance with the SPSS 13.0 software. Values of *P* < 0.05 were considered statistically significant.

## 3. Results

### 3.1. Inhibition of SGC7901/VCR and SGC7901 Cell Proliferation by YJHD

**Figure 1:**
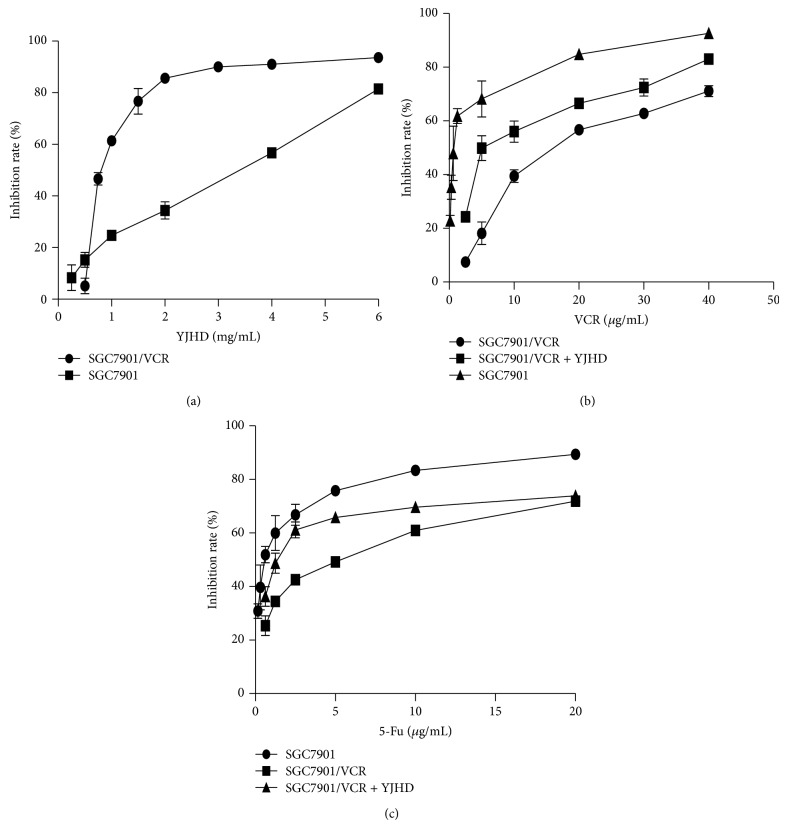
(a) Inhibition ratio of cell proliferation at increasing concentrations of YJHD in the SGC7901/VCR cell line. (b), (c) Cell inhibition ratios at increasing concentrations of VCR, 5-Fu, and VCR combined with 0.5 mg/mL of YJHD in SGC7901/VCR cells. The cell inhibition ratio was determined by the CCK-8 assay.

SGC7901/VCR and SGC7901 cells were treated with 0–6 mg/mL YJHD, and the results demonstrate the dose- and time-dependent inhibition of proliferation by YJHD (Figure 1(a)), inducing a greater than 90% reduction in the number of cells at the highest concentration (6 mg/mL). To evaluate whether YJHD increases the sensitivity of chemotherapeutic agents in an MDR gastric cancer cell line in vitro, we evaluated a nontoxic dose of YJHD combined with various concentrations of VCR and 5-Fu. The concentrations of the chemotherapeutic agents were selected according to their peak plasma concentrations in vivo: 40, 20, 10, 5, 2.5, 1.25, and 0.625 *μ*g/mL. The concentrations of YJHD were 6, 4, 2, 1, and 0.5 mg/mL. The effects of the low dose of YJHD in combination with the chemotherapeutic agents on cell proliferation were measured using the CCK-8 assay. As shown in Figures 1(b)-1(c), a concentration of 0.5 mg/mL of YJHD increased the sensitivity of SGC7901/VCR cells to the chemotherapeutic agents and, to some extent, reversed MDR. The chemosensitivity of MDR cells to the chemotherapy drugs was measured by comparing IC_50_ values, expressed as RF. The IC_50_ values of YJHD were 2.71 ± 0.13 and 0.86 ± 0.046 mg/mL, respectively, for the SGC7901 and SGC7901/VCR cell lines (Table 1). At less than or equal to 0.5 mg/mL, YJHD did not cause any cytotoxicity to SGC7901/VCR cells. Therefore, 0.5 mg/mL was chosen as the working concentration in the subsequent experiments. As expected, the SGC7901/VCR cells were more resistant to VCR. The IC_50_ values for 5-Fu and VCR in SGC7901 cells were 0.63 ± 0.073 and 0.84 ± 0.08 *μ*g/mL, whereas the IC_50_ values for 5-Fu and VCR were 4.378 ± 0.12 and 17.01 ± 0.32 *μ*g/mL for the SGC7901/VCR cells (Table 1). YJHD dose dependently increased the sensitivity of SGC7901/VCR cells to 5-Fu and VCR (Figures 1(b) and 1(c)). When administered at a nontoxic dose, YJHD shifted the IC_50_ values for 5-Fu and VCR to 1.462 ± 0.085 and 7.29 ± 0.24 *μ*g/mL, with RF values of 2.99 ± 0.10-fold and 2.33 ± 0.10-fold, respectively (Tables 2 and 3). However, treatment with YJHD did not significantly affect the drug sensitivity of SGC7901 cells.

### 3.2. Apoptosis in SGC7901/VCR Cells Induced by YJHD in Combination with Chemotherapeutic Drugs

**Figure 2:**
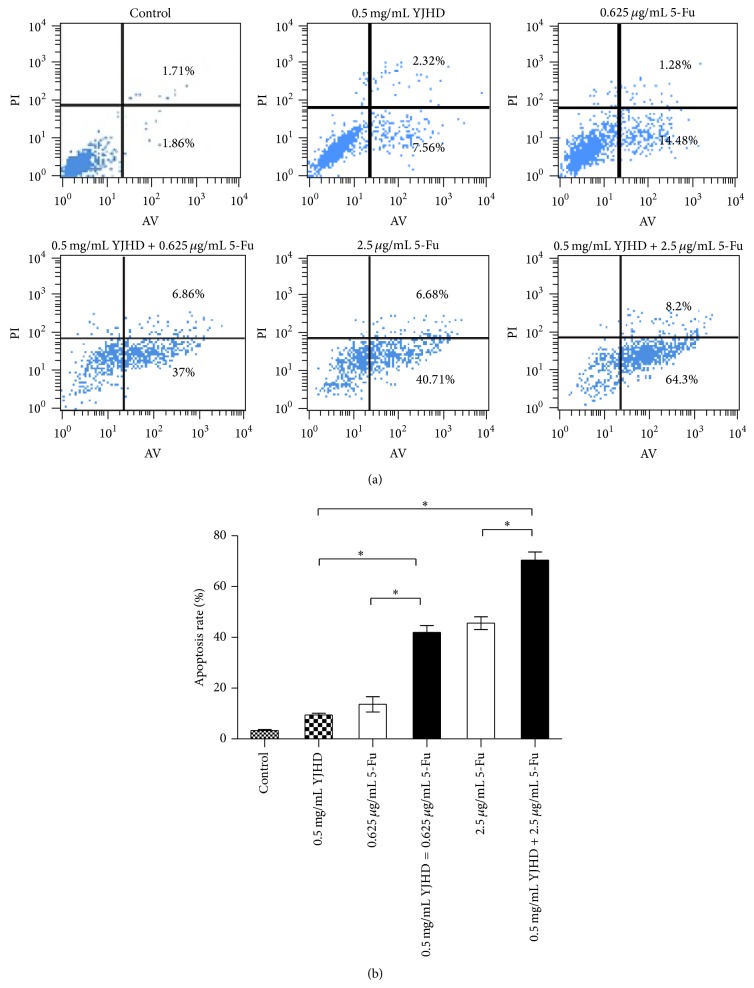
(a) Flow cytometric analysis of apoptosis in SGC7901/VCR cells treated with VCR or 5-Fu, with or without 0.5 mg/mL YJHD, for 48 h. Analysis of the apoptosis ratio. ^*^ *P* < 0.05 versus control. (b) Bar graph of the apoptosis rate. ^*^ *P* < 0.05 versus control.

Apoptosis was measured using flow cytometry. The apoptosis ratios in the control group were 7.39 ± 1.25% after a 48 h treatment with 0.5 mg/mL YJHD. The apoptosis ratios for the YJHD and 5-Fu groups were significantly increased compared to the 5-Fu single-drug group (*P* < 0.05). These results indicate a dose-dependent effect on apoptosis in SGC7901/VCR cells (*P* < 0.05; Figure 2), confirming the apoptotic and inhibitory effects of a low dose of YJHD when administered in combination with 5-Fu on the growth of MDR gastric cancer SGC7901/VCR cells.

### 3.3. Enhancement of 5-Fu-Induced S-Phase Arrest in SGC7901/VCR Cells by YJHD

**Figure 3:**
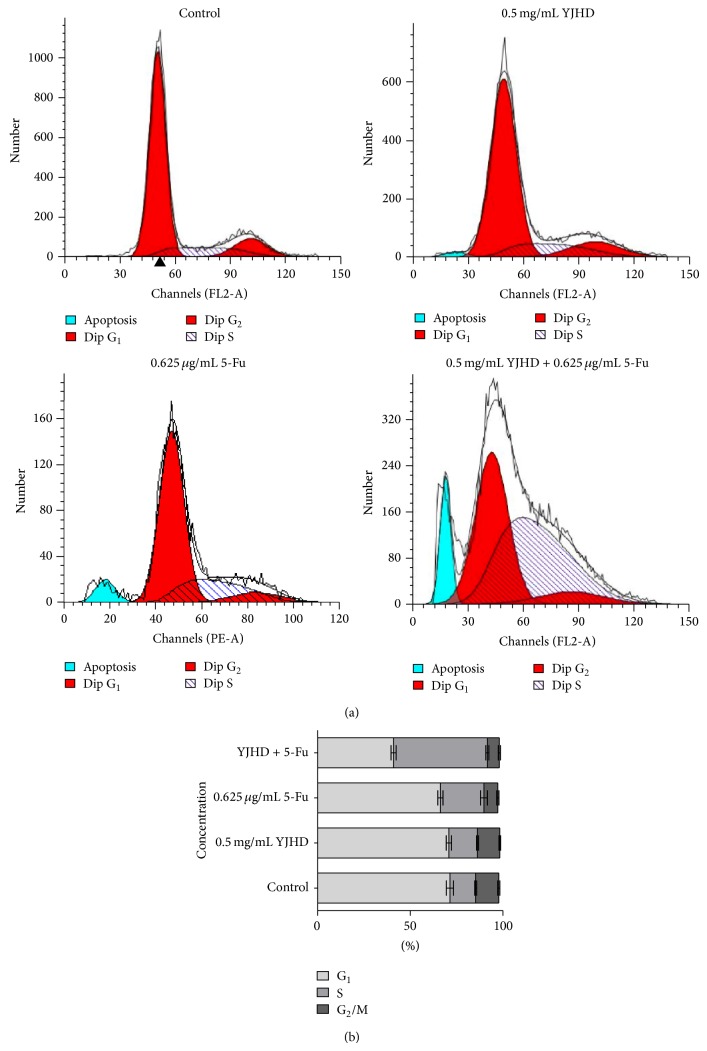
(a) Cells were cultured with 5-Fu, with or without YJHD (0.5 mg/mL), for 48 h and then fixed and stained with PI. The DNA content was analyzed by flow cytometry. G_0_/G_1_, G_2_/M, and S indicate the cell phases. (b) Each phase was calculated using the ModiFit program. Three duplicated experiments were performed with similar results.

In general, agents that induce cytotoxicity by inhibiting DNA synthesis (e.g., 5-Fu) arrest the cell cycle at S phase [18]. As expected, at concentrations equal to 0.625 *μ*g/mL, 5-Fu-induced S-phase arrest. Although a low dose of YJHD (0.5 mg/mL) did not affect the cell cycle when administered by itself, treatment with YJHD in combination with 5-Fu induced a significant S-phase arrest in up to 50.62 ± 0.81% of the cells (Figure 3). These results suggest that YJHD enhanced 5-Fu-induced S-phase arrest, which may be attributed to its effect on drug resistance.

### 3.4. Influence of YJHD on the Expression of mRNA and Protein Encoding Chemotherapeutic Agent Resistance-Related Genes

**Figure 4:**
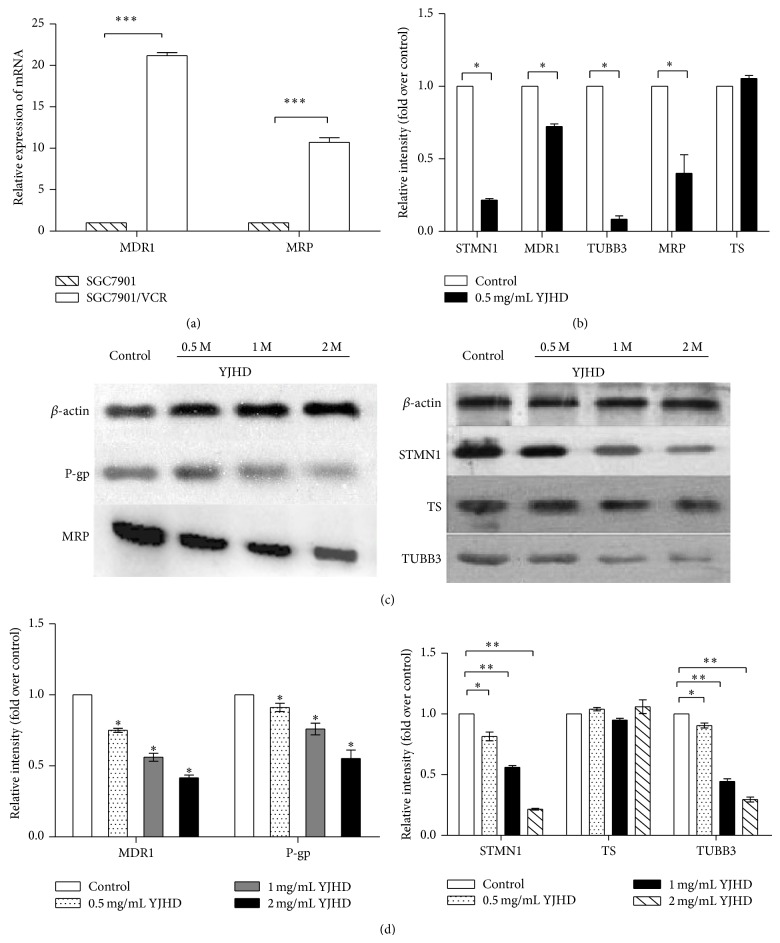
(a) MDR1 and MRP gene expression in SGC7901 and SGC7901/VCR cells. ^***^ *P* < 0.01 versus control. (b) YJHD suppressed the mRNA expression of chemotherapeutic agent resistance-related genes in SGC7901/VCR cells. Fold changes: relative gene expression after treatment with YJHD at 0.5 mg/mL for 48 h compared to control. ^*^ *P* < 0.05 compared with control. (c) Western blot analysis of STMN1, TS, TUBB3, MRP, and P-gp in control cells and cells treated with YJHD at 0.5 mg/mL, 1 mg/mL, and 2 mg/mL. (d) Bar graph of the densitometric values of the bands corresponding to MRP, P-gp, STMN1, TS, and TUBB3 normalized relative to *β*-actin. ^*^ *P* < 0.05 versus control, ^**^ *P* < 0.01 versus control.

To investigate the mechanisms underlying the synergistic interaction between a low dose of YJHD and 5-Fu or VCR, we hypothesized that YJHD might affect the expression of chemotherapeutic agent resistance-related genes, that is,* MDR1*,* MRP*,* TS*,* STMN1*, and* TUBB3*, by influencing their sensitivity to 5-Fu and VCR. We first found that the gene expression of MDR1 and MRP in SGC7901/VCR cells is higher than that in SGC7901 cells. After incubating the cells with YJHD at their respective IC_40_ values for 48 h, the levels of mRNA expression of these genes in SGC7901/VCR cells were assessed by real-time PCR. As shown in Figure 3, the levels of MDR1, MRP, TUBB3, and STMN1 were significantly downregulated by YJHD treatment, though YJHD did not downregulate the expression of TS. Similar results were obtained by western blotting, indicating that the expression levels of P-gp, MRP, TUBB3, and STMN1 were strongly positive in SGC7901/VCR cells for 48 h. The expression levels were slightly positive in cells treated with 0.5 mg/mL of YJHD for 48 h (Figure 4), with very little effect on TS. These results demonstrate that YJHD decreased the expression of P-gp, MRP, TUBB3, and STMN1 in SGC7901/VCR cells and increased the antitumor effects of chemotherapeutic drugs by inhibiting their expression.

The stable knockdown of oncogenic STMN1, TUBB3, MRP, and MDR1 by siRNA significantly reversed the drug resistance of SGC7901/VCR cells in vitro.

**Figure 5:**
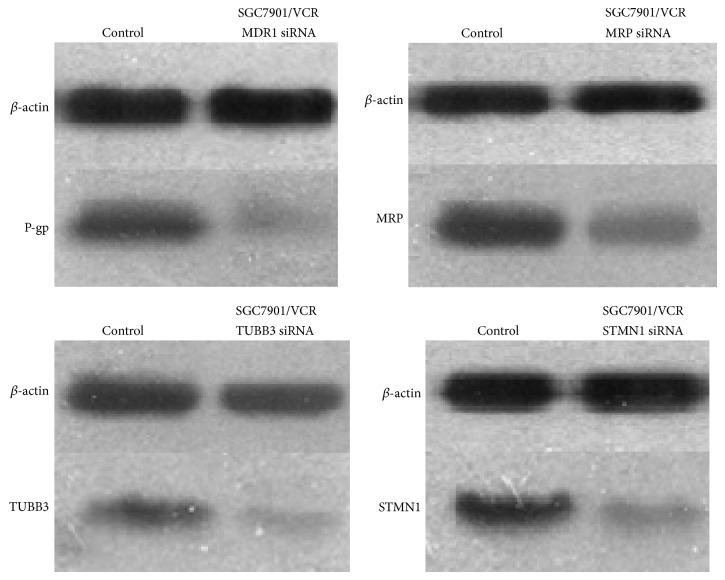
STMN1, MDR1, TUBB3, and MRP expression in stably transfected cells. The transfected cells were identified by western blotting. *β*-actin was used as an internal control.

Thus, to investigate the possible effects of knockdown of these genes in vitro, all four were downregulated in the SGC7901/VCR cell line (Figure 5). All cells were evaluated by comparison with IC_50_ values using a CCK-8 assay, revealing that the proliferation of STMN1-, MDR1-, MRP-, TUBB3-knockdown cells was significantly diminished compared with the negative-control cells. Moreover, these knockdown cells exhibited significantly higher sensitivity to vincristine than the control cells (*P* < 0.01; Table 4).

## 4. Discussion

Although chemotherapy is an important treatment modality for gastric cancer, because of MDR, its effectiveness is significantly reduced as metastasis and recurrence ratios increase. Thus, the reversal of MDR and increase in the sensitivity to chemotherapeutic agents are very important aspects of tumor therapy. YJHD is a traditional Chinese medicine that is an extract comprising 12 species of medicinal plants. Several components have recently been reported to exert antiproliferative effects on several cancer cell lines in vivo and in vitro [14–16], including lung cancer, breast cancer, and colon cancer cells, with several components reducing resistance in breast cancer cell lines [14]. Our previous studies confirmed that YJHD has an antiproliferative effect on the SGC7901 human gastric cancer cell line in vitro [19]. In the present study, we explored the effects of YJHD in an MDR gastric cancer cell line and investigated the underlying mechanism of action. The results of the CCK-8 assay and flow cytometry demonstrated that YJHD dose dependently inhibited proliferation and induced apoptosis in SGC7901/VCR cells (*P* < 0.05). According to the peak plasma concentrations of chemotherapeutic agents in vivo, we observed inhibitory effects of the combination of a low dose of YJHD with 5-Fu and VCR on cell proliferation, showing that low-dose YJHD increased the inhibitory effects.

The influence of YJHD on the expression of intracellular cyclins can cause cell cycle arrest and induce apoptosis in SGC7901 cells [19]. However, our experimental results indicate that a low dose of YJHD does not affect the cell cycle. Our results also suggested that 5-Fu dose dependently induces cell cycle arrest at S phase in SGC7901/VCR cell lines. Moreover, a low concentration of YJHD (0.5 mg/mL) enhanced the sensitivity of SGC7901/VCR cells to 5-Fu (cell cycle arrest at S phase).

Furthermore, because several drug-metabolizing genes have been shown to be prognostic markers for gastric cancer therapy, we examined the additional influence of YJHD on their mRNA and protein expression.

Microtubules are composed of tubulin dimers and interact with a variety of microtubule-associated proteins [20]. Studies in lung cancer have shown that high expression levels of brain-specific TUBB3 are associated with vincristine resistance in a preclinical setting, which has been further confirmed in patients with advanced NSCLC [21]. STMN1 has been shown to regulate the dynamics of the microtubules that compose the mitotic spindle, and STMN1 overexpression leads to the resistance to antimicrotubule agents [22]. A decreased sensitivity of breast cancer cells to* Vinca* alkaloids and Taxol in response to the overexpression of the microtubule-associated protein STMN1 was reported by Alli et al. [22], and a high TS expression level was reported to be associated with resistance to 5-Fu therapy and a poor clinical outcome [23]. A meta-analysis [24] demonstrated that the TS expression level should be considered one of the most important markers of the 5-Fu response. A previous study demonstrated that P-gp is overexpressed in* H. pylori* positive patients, particularly in patients who do not respond to eradication therapy [5], and a causal link has been reported between MRP and P-gp activity, which may have implications for MDR in tumors in which MRP is overexpressed. Our data also indicate that YJHD downregulated the expression of* MRP*,* TUBB3*,* STMN1*,* MDR1* mRNA, and protein, though the expression of TS was not significantly affected. In this study, we found that the capacity of the Chinese medicine to reverse resistance to fluorouracil is stronger than that of vincristine. Accordingly, we suggest that the drug-induced resistance mechanism of the latter is more complex, with other mechanisms of resistance in addition to an increase in the expression of four genes.

We then used RNAi technology to knock down the expression of these four genes and found that the sensitivity to chemotherapy drugs by multidrug-resistant cells was notably increased, confirming our results.

To our knowledge, the present study is the first to demonstrate the effects of YJHD on an MDR human gastric cancer cell line. YJHD inhibited proliferation, induced apoptosis, circumvented MDR, and increased sensitivity to chemotherapeutic agents in the P-gp-overexpressing gastric cancer cell line SGC7901/VCR. The effects of YJHD may be attributed to the downregulation of the expression of MDR1/P-gp, MRP, TUBB3, and STMN1, thus weakening the level of P-gp-mediated MDR and increasing the sensitivity of SGC7901/VCR cells to chemotherapy.

The present study suggests that the use of chemotherapeutic agents in combination with YJHD as a chemosensitizer or adjuvant may be helpful for the treatment of gastric cancer. We also found that chemotherapy together with RNA interference appears to exert a promising therapeutic effect. Although clinical application needs to be evaluated, these findings provide an intriguing possibility for future cancer therapy.

## Figures and Tables

**Table 1:** IC_50_ values (*μ*g/mL) of anticancer drugs in gastric carcinoma cells.

Cell line	5-Fu (*μ*g/mL)	VCR (*μ*g/mL)	YJHD (mg/mL)
SGC7901	0.63 ± 0.073	0.84 ± 0.08	2.71 ± 0.13
SGC7901/VCR	4.378 ± 0.12	17.01 ± 0.32	0.86 ± 0.046
RI	6.94	20.25	

Reversal factors (RFs) were calculated according to the following equation: RFs = IC_50_ antitumor drug alone/IC_50_ antitumor drug + modulator.

**Table 2:** Magnitude of the reversal of multiple drug resistance by a low dose of YJHD.

Cell line	5-Fu (*μ*g/mL)	5-Fu + 0.5 M YJHD (*μ*g/mL)
SGC7901/VCR	4.378 ± 0.12	1.462 ± 0.085
RF (5-Fu IC_50_/5-Fu + YJHD IC_50_)		2.99 ± 0.10

**Table 3:** Magnitude of the reversal of multiple drug resistance by a low dose of YJHD.

Cell line	VCR (*μ*g/mL)	+0.5 M YJHD (*μ*g/mL)
SGC7901/VCR	17.01 ± 0.32	7.29 ± 0.24
RF (VCR IC_50_/VCR + YJHD IC_50_)		2.33 ± 0.10

**Table 4:** Effect of siRNAs on the sensitivity of SGC7901/VCR tumor cells to chemotherapeutic drugs (vincristine) after transfection.

Drug	IC_50_ (*μ*g/mL)			

Vincristine	STMN1	TUBB3	MDR1	MRP
	siRNA	siRNA	siRNA	siRNA

17.01 ± 0.32	7.13 ± 1.15^*^	5.05 ± 0.29^*^	1.77 ± 0.38^*^	2.69 ± 0.29^*^

Cells were plated in 96-well multiwell plates. After 24 hr of transfection, the cells were exposed to various concentrations of cytotoxic drugs for 2 days. After CCK-8 was added, the absorbance in individual wells was determined at 450 nm, and the IC_50_ was calculated.

The results represent the mean ± SD of three separate experiments.

^*^ *P* < 0.05 compared with control.
